# Matrons in Council

**Published:** 1917-10-13

**Authors:** 


### THE MATRONS' AND SISTERS' DEPARTMENT.

### MATRONS IN COUNCIL.

### X. Personality and Progress.

There can be no doubt that the personality of the nurse is a matter of first importance. However desirable the passing of examinations in theory may be, this should never be allowed to be placed in the forefront, and when we have occasion to look round and weigh up personality *versus* high examination marks, we easily make our choice when it is a question of whom we would like to nurse our friends. It is obvious to all thinking people that the Dominions and America have outpaced us in the matter of education, and it behoves us to follow their lead. Nevertheless, our best type of nurse is still hard to beat, especially in the ward and home.

Our house needs setting in order, and we must see to it that it is done; but the question upon which there is disagreement is whether the present is the right time in which to grapple with so big and far-reaching a problem. Speaking from personal experience, the most we can do at present is to "carry on" under conditions which change from day to day. Nurses are passing almost without exception into some branch of military work on completing their training, and the larger number of senior sisters and nurses have been doing so since 1914. This of necessity means that those who are responsible for training our future nurses are unable to bring to the work the ripe experience of pre-war days. Add to this the inevitable spirit of unrest which at present permeates all work, and it must be conceded that it is largely a question of doing one's best with the material to our hand. The general hospitals are, and have been, more or less sacrificed to the military since 1914.

To sum up, it is evident that the whole question of nurses and nursing must be reorganised root and branch. But it is a question which must be tackled drastically, and with the help of the many excellent members of the profession who are at present working in other spheres, rather than institute a partial measure of improvement without the help of all.

> Greatly begin, if you have time
> But for one line—be that sublime,
> *Not* failure, but low aim is crime.

C. M. E.

### XI. Standardise Teaching and Improve Pay.

The efficient training of probationers rests very largely on the conditions of supply and demand. In immediate pre-war times nursing appealed to very few women of even fair education. Matrons had to take candidates who they knew would have been better employed as shop-girls or domestic servants, and experienced ward sisters, realising they must have hands if they could not get heads, made the best of the material at hand. Given a good supply of candidates from whom to select, and freely rejecting unsuitable probationers after their trial period, no doubt the status of nurses would be raised, and the profession take its rightful place in the first rank of women's work. To do this, teaching must be in a measure uniform, allowing for different conditions in different neighbourhoods, but above all probationers must be paid at a higher rate during their training. They cannot gain a certificate in a good general hospital before they are twenty-four. In what other profession do women have to wait so long before they can earn a livelihood?

It is not the long hours or the strenuous work, but the lean years which stretch before her, which cause many a young probationer to lose heart. Cannot we learn from our Church, where many an able man is lost on account of inadequate remuneration? The time is coming, and not too soon, when the pay of school teachers will be increased, with the hope of attracting a better type to teach our children, and hospitals must come into line, or lose the best material. Many nursing committees realise this, but unless all are speeded up, nursing will be left to those who think it is a fine thing to don a uniform, forgetting that hospitals are built for the care of the sick. Let us hope that the College of Nursing will help to level us up to a higher standard.

Dartmoor.

### XII. The Responsibility of Matron and of Nurse.

There is doubtless very much lacking in the upbringing of nurses of the present day. These are times of special difficulty, and in the stress of work and the shortage of senior sisters the training is bound to suffer, but I do not think the failures are due solely to war conditions; they existed before. Doubtless many things need altering and improving—the education of nurses, the long hours on duty, the insufficient salaries, and, above all, the "tone" of the nursing profession as a whole.

But I think the writer of "Sparks from a Nurse's Anvil" has gone to the root of the matter when she mentions the attitude of some matrons towards their nurses. If it is true that "the character of a nation is moulded by the character

☛ Previous articles appeared in The Hospital of August 4, 11, 18, 25, Sept. 1, 15, 22, and 29, and Oct. 6.

of its individuals," so, surely, is the character and tone of the nursing profession moulded by individual nurses, and it is therefore of vast importance to select the right women for training. And this responsibility—and a great one it is—rests mainly with the matrons. I know it is very difficult to pick and choose when the need is so great and urgent, but it seems to me a vital necessity that only those candidates should be accepted who have at least some serious and worthy motive in entering the profession.

Then, again, it rests with the matrons chiefly not only to select the right material, but to try to hold up before their nurses a high ideal, not only of professional efficiency, but, what is equally important, of personal character. Let them teach their nurses to "aim high."

Your correspondent expresses what I have always felt very strongly—matrons would do well to remember that the very meaning of their title indicates one of their chief duties, and also one of their chief joys; it implies that they should be as a "mother" to their nurses.

As an old matron I think I can safely say that there is no need to place oneself on a pedestal to gain the respect of one's nurses. If a matron will only let her nurses feel that she is human, and that she understands and sympathises with their difficulties and joys and sorrows and ambitions, she will gain their respect and loyalty, and also their love. The nurses will learn to look on their training-school as their home, and will make it a point of honour to uphold its good name and to carry its traditions into their various new spheres of work.

A Matron of Many Years' Experience.

